# Prognostic value of lymphocyte to monocyte ratio in patients with esophageal cancer: a systematic review and meta-analysis

**DOI:** 10.3389/fonc.2024.1401076

**Published:** 2024-11-26

**Authors:** Yongqi Han, Song Zheng, Yijing Chen

**Affiliations:** ^1^ Department of Oncology, The Fourth School of Clinical Medicine, Zhejiang Chinese Medical University, Hangzhou, China; ^2^ Department of Oncology, Hangzhou First People’s Hospital, Hangzhou, China; ^3^ Department of Oncology, Hangzhou Cancer Hospital, Hangzhou, China; ^4^ Key Laboratory of Clinical Cancer Pharmacology and Toxicology Research of Zhejiang Province, Hangzhou First People’s Hospital, Hangzhou, China; ^5^ Department of Oncology, Zhejiang University School of Medicine, Hangzhou, China

**Keywords:** lymphocyte, monocyte, prognosis analysis, esophageal cancer, meta-analysis

## Abstract

**Objectives:**

To report the largest systematic review and meta-analysis to evaluate prognostic value of lymphocyte to monocyte ratio (LMR) in patients with esophageal cancer.

**Methods:**

We conducted a systematic literature retrieval via PubMed, Embase, Web of Science, and Cochrane until December, 2023 for studies which evaluated the prognostic value of LMR in patients with esophageal cancer. Outcomes measured were overall survival (OS), disease-free survival (DFS), relapse-free survival (RFS), and progression-free survival (PFS).

**Results:**

11 studies including 3,377 patients with esophageal cancer were included for meta-analysis. Meta-analysis demonstrated that OS (HR: 1.65; 95% CI: 1.19, 2.31; *P* = 0.003) and DFS (HR: 1.48; 95% CI: 1.09, 2.01; *P* = 0.01) were significantly shorter in the low LMR group compared with the high LMR group. In addition, meta-analysis revealed a similar PFS (HR: 1.58; 95% CI: 1.00, 2.51; *P* = 0.05) and RFS (HR: 1.17; 95% CI: 0.93, 1.46; *P* = 0.18) in the two groups. Subgroup analysis found that the predictive value of LMR for OS remained significant in resectable and unresectable esophageal cancers, and in studies with follow-up ≥24 months and < 24 months. Subgroup analysis based on treatment methods found that the prognostic value of LMR was significant for both patients who received PD-1/PD-L1 inhibitors and those who did not receive PD-1/PD-L1 inhibitors. However, subgroup analysis based on LMR threshold found that the significance remained in studies with LMR threshold<3.5 (HR: 2.09; 95% CI: 1.13, 3.87; P = 0.02) but disappeared in studies with LMR threshold ≥ 3.5 (HR: 1.39; 95% CI: 0.93, 2.07; P = 0.11).

**Conclusions:**

Low LMR is associated with poor prognosis in patients with esophageal cancer. Due to the simple availability and low cost of routine blood tests in clinical practice, LMR can be widely used to assess prognosis and construct risk prediction models for patients with esophageal cancer.

**Systematic review registration:**

PROSPERO, identifier CRD42024509796.

## Introduction

Esophageal cancer is one of the most common malignant tumors in the world. According to the latest global cancer statistics report in 2020, there are 604,100 new cases of esophageal cancer in the world, and 544,000 new deaths, ranking 7th in the world in terms of incidence and 6th in terms of overall mortality (1). The 5-year overall survival rate of esophageal cancer in the world is 10% ~ 30% (2), and the overall prognosis is not good. Esophageal cancer is a malignant invasive disease characterized by a high rate of lymph node metastasis and easy recurrence after treatment. China is a high incidence area of esophageal cancer, mainly esophageal squamous cell carcinoma (3). The main treatment for esophageal cancer is radical resection of esophageal lesions combined with lymph node dissection (4). However, due to the atypical early symptoms of esophageal cancer and its easy occurrence of lymph node metastasis, tumors are usually found in the middle and late stages. Even in the early stages of the disease, most patients with esophageal cancer still die from regional recurrence or distant metastasis.

Unlike other digestive system tumors, esophageal cancer lacks blood biomarkers for predicting prognosis and evaluating tumor sensitivity to treatment, as well as biomarkers for risk stratification. Systemic inflammatory response is one of the recognized features of malignant tumors. The occurrence of systemic inflammatory response is related to the occurrence and development of tumors, and the inflammatory response of host cells to tumors has been shown to inhibit apoptosis and promote DNA damage, resulting in excessive proliferation and premature metastasis of tumors (5). Previous studies calculated neutrophil to lymphocyte ratio (NLR), platelet to lymphocyte ratio (PLR), and lymphocyte to monocyte ratio (LMR) based on human inflammatory cells (6, 7).

A large number of relevant studies suggest that LMR is associated with the prognosis of esophageal cancer (8–20). However, its value in evaluating the prognosis of esophageal cancer is still unclear, and the conclusions of different studies are not completely consistent, and there is a lack of comprehensive evidence-based medical evidence. Therefore, this study conducted a meta-analysis of the relationship between LMR level and prognosis in patients with esophageal cancer, in order to systematically evaluate the predictive effect of LMR on prognosis in patients with esophageal cancer.

## Methods

### Literature search

This meta-analysis was conducted in strict adherence to the PRISMA (Preferred Reporting Items for Systematic Reviews and Meta-Analysis) 2020 statement (21) and has been prospectively registered in the PROSPERO (CRD42024509796). We conducted a systematic literature search via PubMed, Embase, Web of Science, and Cochrane up to December, 2023 for studies that evaluated the role of LMR in the prognosis of esophageal cancer. We searched the literature through the following terms: “esophageal neoplasms”, “lymphocytes”, and “monocytes”. The detailed search strategies are as follows: ((((“Lymphocytes”[Mesh]) OR (((((Lymphocyte) OR (Lymphoid Cells)) OR (Cell, Lymphoid)) OR (Cells, Lymphoid)) OR (Lymphoid Cell))) AND ((“Monocytes”[Mesh]) OR (Monocyte))) AND (ratio)) AND ((“Esophageal Neoplasms”[Mesh]) OR (((((((((((((((((Esophageal Neoplasm) OR (Neoplasm, Esophageal)) OR (Esophagus Neoplasm)) OR (Esophagus Neoplasms)) OR (Neoplasm, Esophagus)) OR (Neoplasms, Esophagus)) OR (Neoplasms, Esophageal)) OR (Cancer of Esophagus)) OR (Cancer of the Esophagus)) OR (Esophagus Cancer)) OR (Cancer, Esophagus)) OR (Cancers, Esophagus)) OR (Esophagus Cancers)) OR (Esophageal Cancer)) OR (Cancer, Esophageal)) OR (Cancers, Esophageal)) OR (Esophageal Cancers))). Furthermore, we manually screened the bibliography lists of all included studies. Two authors (Han & Chen) retrieved and assessed eligible articles independently. Any differences in literature retrieval were resolved by discussion.

### Inclusion and exclusion criteria

Eligible studies met these criteria: (1) Utilized a randomized controlled trial, cohort, or case-control de-sign; (2) Focused on esophageal cancer patients; (3) Examined the prognostic significance of the LMR; (4) Included survival outcomes such as overall survival (OS), disease-free survival (DFS), or progression-free survival (PFS); (5) Provided comprehensive data for risk ratio (RR), odds ratio (OR), or hazard ratio (HR) analysis. We excluded study protocols, unpublished studies, non-original studies (including letters, comments, abstracts, correction, and reply), studies without sufficient data, and reviews. We excluded study protocols, unpublished studies, non-original studies (including letters, comments, abstracts, correction, and reply), studies without sufficient data, reviews and studies with the Newcastle-Ottawa Scale (NOS) scores below 6.

### Data abstraction

Data extraction was performed by two authors separately, and all data were summarized in independent Excel tables. After data extraction, the two authors summarized and checked the data. If there were any disagreements, they were resolved through discussion. If no consensus could be reached, the other author made the decision. We abstracted following information from eligible studies: first author name, published year, research period, study region, study design, research population, sample size, age, gender, tumor size, follow-up, LMR threshold, OS, PFS, DFS, and RFS. If the research data is insufficient, corresponding authors were contacted for full data if available.

### Quality evaluation

The NOS served as the evaluation tool for determining the quality of the included cohort studies (22), and studies with 7-9 points were considered as high quality (23). Studies with NOS scores below 6 were not included for quantitative analysis. Two authors severally assessed the quality of all included studies, and any disagreement was settled by discussion.

### Statistical analysis

A meta-analysis was performed using Review Manager (RevMan) version 5.4.1. Hazard ratios (HRs) were employed to synthesize survival data, and these metrics were reported alongside 95% confidence intervals (CIs) for precision. To assess the heterogeneity across the outcomes, the chi-squared (χ^2^) test (Cochran’s Q) and the inconsistency index (*I* ^2^) were utilized (24). χ^2^ *P* value less than 0.1 or *I* ^2^ more than 50% were regarded as high heterogeneity. The fixed-effects and random-effects models were applied to calculate the total HR for outcomes with significant heterogeneity (χ^2^ *P* value less than 0.1 or *I* ^2^ more than 50%). Or else, only the fixed-effects model was used. In addition, we performed subgroup analyses for outcomes with five or more studies included to evaluate the possible confounders, if data were sufficient. Besides, we conducted sensitivity analysis to assess the influence of every included study on the total HR for results with significant heterogeneity. Moreover, we assessed the potential publication bias by producing funnel plots through Review Manager 5.4.1 edition as well as through performing Egger’s regression tests (25) through Stata 15.1 edition (Stata Corp, College Station, Texas, USA). *P* value < 0.05 was considered as statistically significant publication bias.

## Results

### Literature retrieval, study characteristics, and baseline

**Figure 1:**
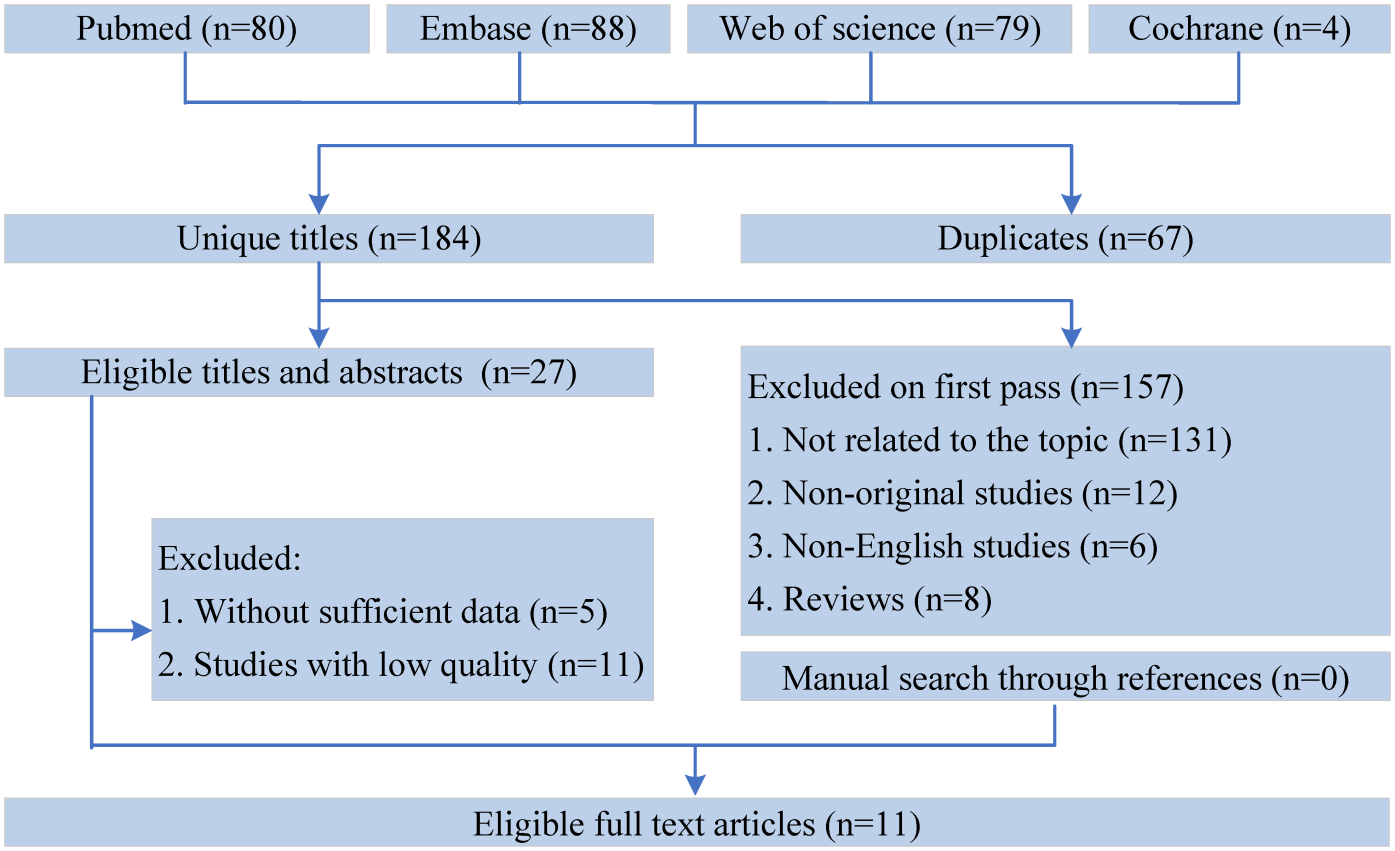
Flowchart of the systematic search and selection process.

**Figure 1** shows the flowchart of the literature retrieval and selection process. A total of 1351 related studies in PubMed (n = 80), Embase (n = 88), Web of Science (n = 79), and Cochrane (n = 4) were identified via systematically literature search. After removing duplicate studies, a total of 184 titles and abstracts were evaluated. Eventually, 11 cohort studies were included for meta-analysis (13–20, 26–28). **Table 1** presents the characteristics and quality evaluation of each eligible cohort study.

**Table 1:** Baseline characteristics of include studies and methodological assessment.

Authors	Study period	Region	Study design	Population	No. of patients (male/female)	Mena/median age (years)	Mean/median tumor length (cm)	LMR threshold	Quality score
Chen (20)	2020	China	Retrospective cohort	M0 (stage I–III) esophageal squamous cell carcinomas	178 (139/39)	NA	NA	3.88	8
Chen (16)	2008-2018	China	Prospective cohort	Newly diagnosed superficial esophageal squamous cell carcinomas (SESCC) (clinical stage Tis or T1N0M0)	156 (152/4)	52.2	3.6	4	7
Han (26)	2007-2008	China	Retrospective cohort	Resectable esophageal squamous cell carcinoma	218 (177/41)	60.5	NA	2.57	8
Hirahara (27)	2006-2014	Japan	Retrospective cohort	Patients who underwent potentially curative esophagectomy with R0 resection for histologically verified esophageal squamous cell carcinoma	65 (62/3)	65.8	4.9	4	8
Huang (14)	2002-2017	China	Retrospective cohort	Elderly esophageal squamous cell carcinoma patients who received radiotherapy with or without chemotherapy	166 (117/49)	NA	NA	1.68	8
Li (28)	2010-2014	China	Retrospective cohort	Advanced esophageal cancer who underwent concurrent chemoradiotherapy	204 (171/33)	65.8	4.8	3.03	8
Ma (15)	2017-2021	China	Retrospective cohort	Unresectable esophageal squamous cell carcinoma who receive first-Line PD-1/PD-L1 inhibitors combined with chemotherapy	81 (74/7)	62.5	NA	2.5	8
Qi (13)	2019-2022	China	Prospective cohort	Esophageal squamous cell carcinoma patients treated with neoadjuvant chemoradiotherapy and pembrolizumab	51 (44/7)	62	4.7	3.36	8
Shang (19)	2005-2015	China	Retrospective cohort	ESCC who underwent radical esophagectomy	1,883	60	NA	3.83	8
Zhao (18)	2012-2015	China	Retrospective cohort	Newly diagnosed locally advanced esophageal squamous cell carcinoma (LA-ESCC) who received neoadjuvant chemoradiotherapy	87 (73/14)	57.69	NA	3.73	8
Zhi (17)	2013-2016	China	Retrospective cohort	Non-surgical esophageal squamous cell carcinoma patients who underwent radiotherapy	193 (108/85)	71.6	NA	NA	8

### OS

**Figure 2:**
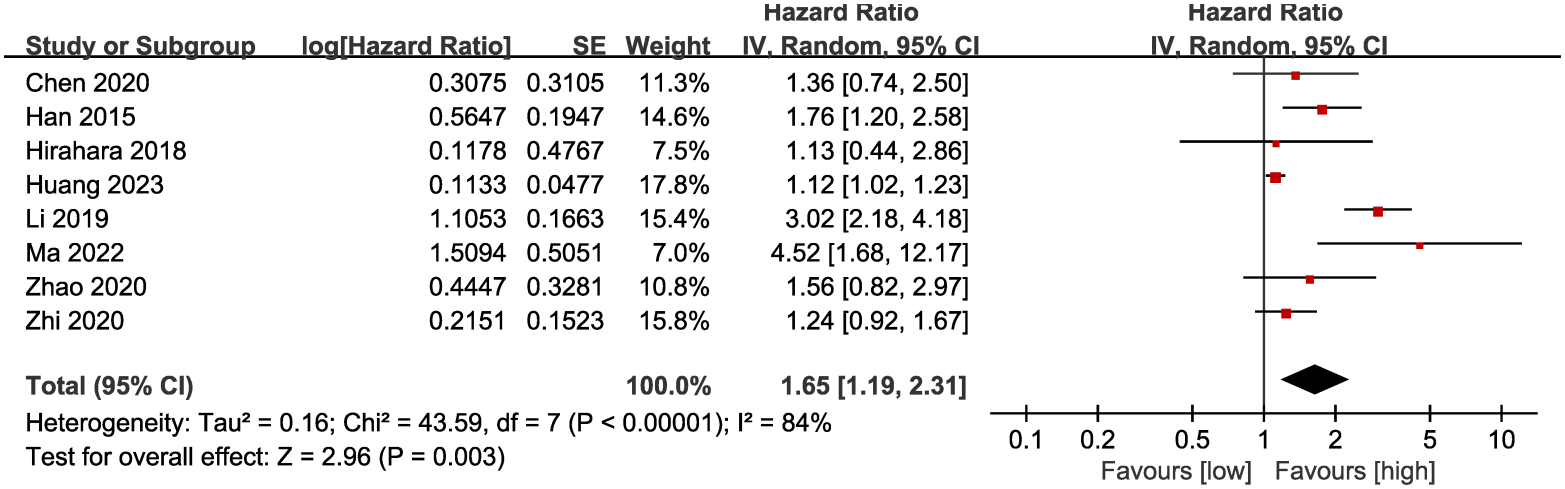
Forest plots of OS.

Results of OS were synthesized from 8 cohort studies (14, 15, 17, 18, 20, 26–28), and meta-analysis revealed a significantly shorter OS in the group with low LMR compared with the group with high LMR (HR: 1.65; 95% CI: 1.19, 2.31; *P* = 0.003). A significant heterogeneity was observed (*I* ^2^ = 84%, *P* <0.00001) (**Figure 2**). Results of the fixed-effects model outcome were provided in the **Supplementary File**.

**Figure 3:**
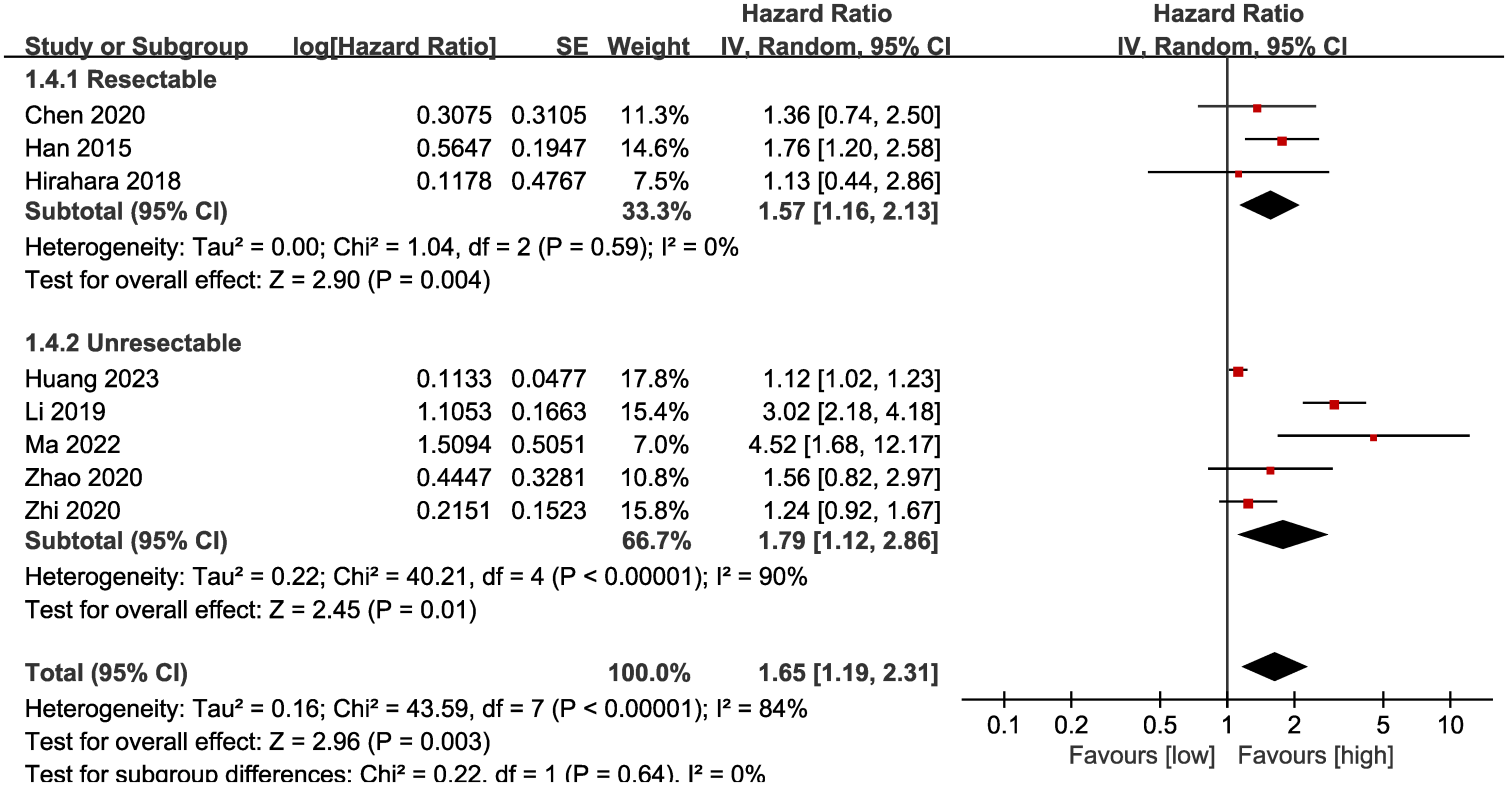
Subgroup analysis of OS based on the types of esophageal cancer.

**Figure 4:**
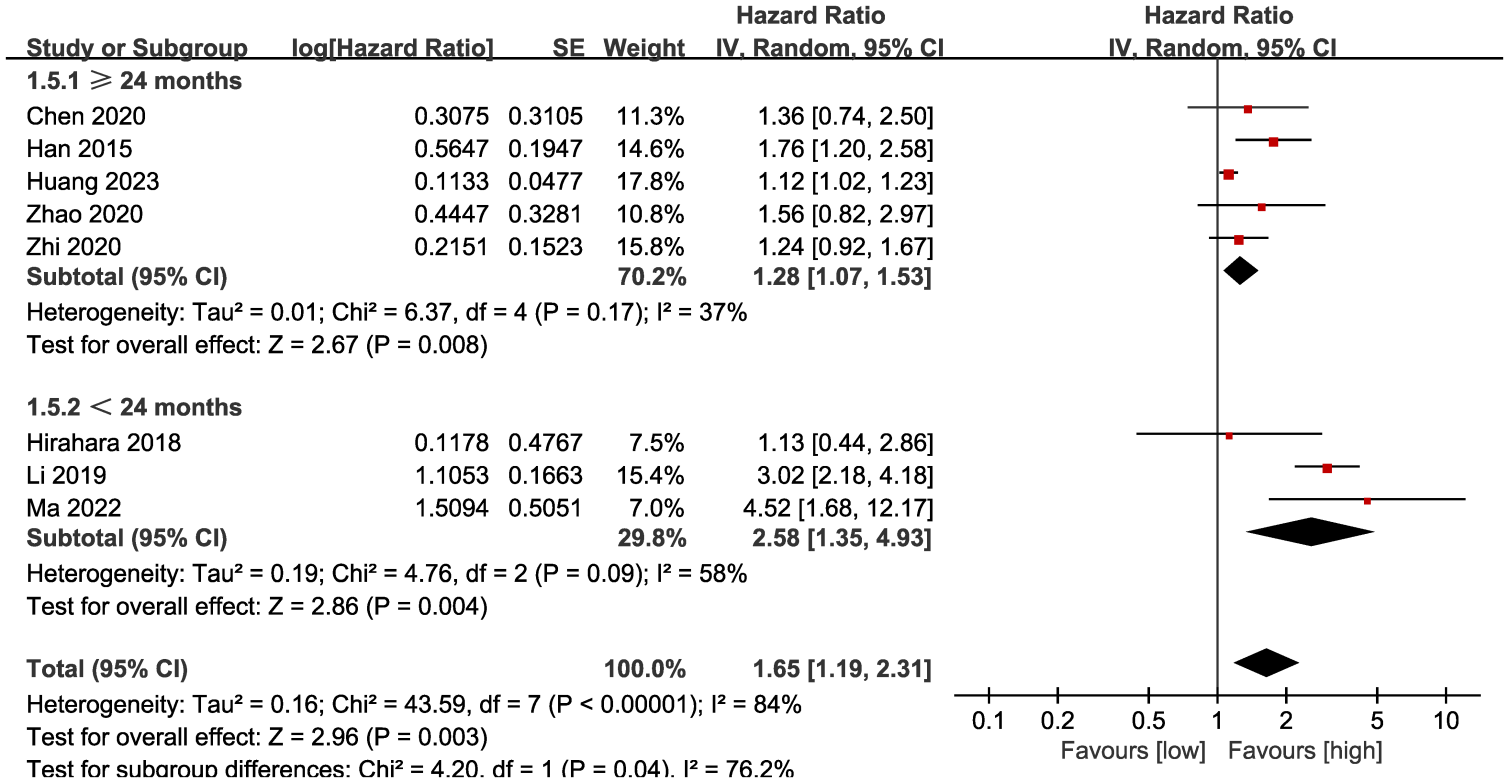
Subgroup analysis of OS based on the duration of follow-up.

**Figure 5:**
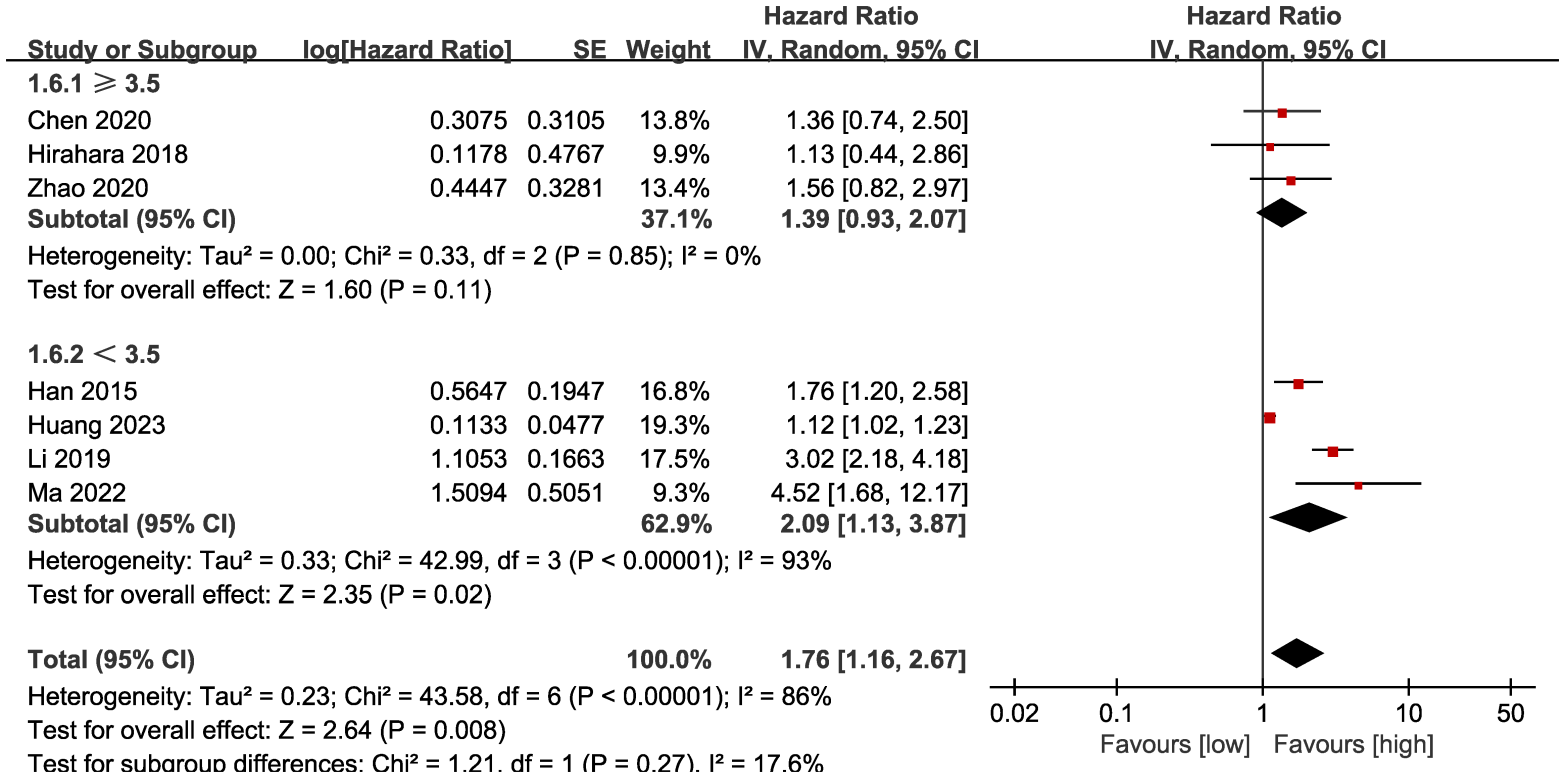
Subgroup analysis of OS based on the LMR threshold.

**Figure 6:**
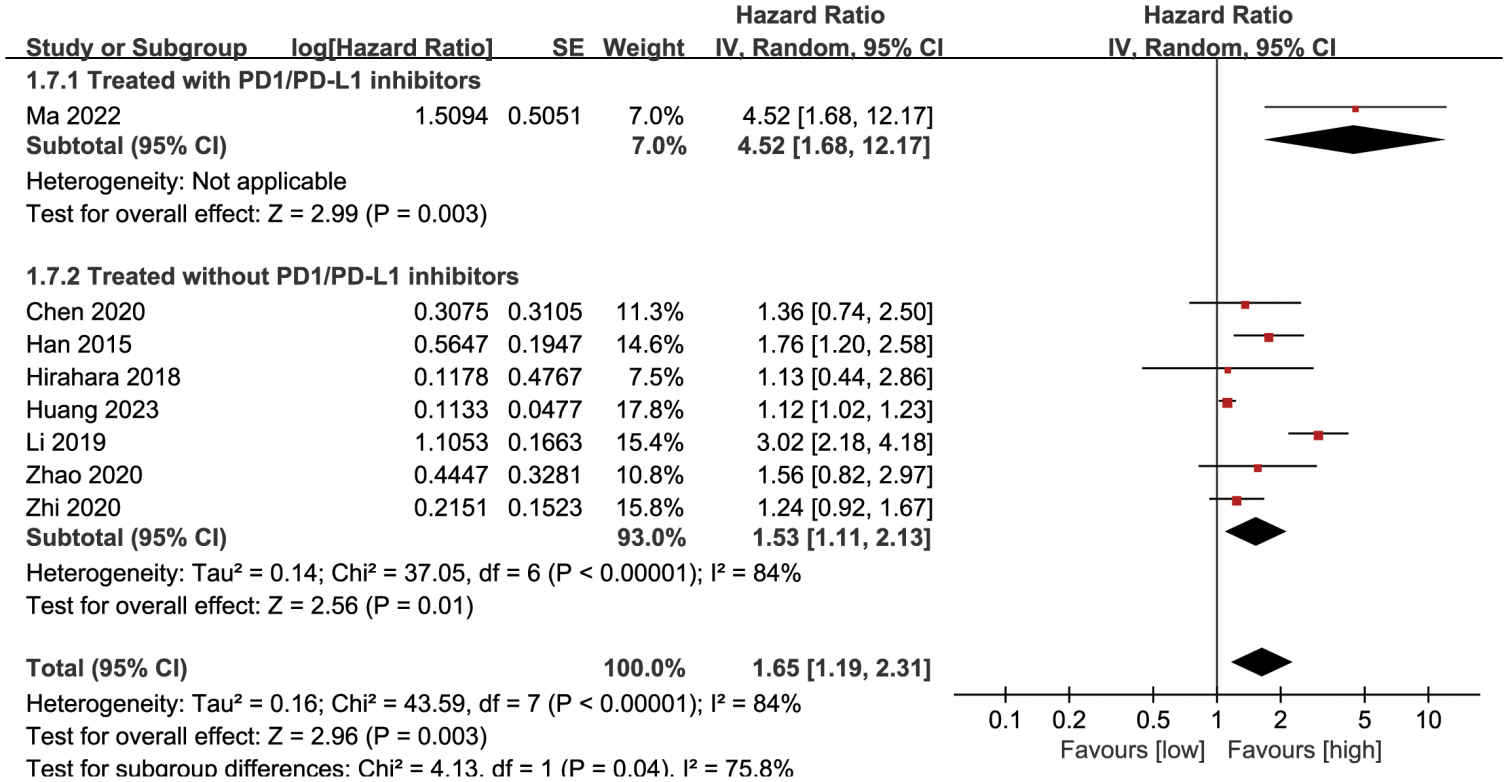
Subgroup analysis of OS based on treatments.

Subgroup analysis based on the types of esophageal cancer found that the significance remained in resectable (HR: 1.57; 95% CI: 1.16, 2.13; *P* = 0.004) and unresectable (HR: 1.79; 95% CI: 1.12, 2.86; *P* = 0.01) esophageal cancer (**Figure 3**). In addition, subgroup analysis based on duration of follow-up found that the significance remained in studies with follow-up ≥ 24 months (HR: 1.28; 95% CI: 1.07, 1.53; *P* = 0.008) and studies with follow-up<24 months (HR: 2.58; 95% CI: 1.35, 4.93; *P* = 0.004) (**Figure 4**). Furthermore, subgroup analysis based on LMR threshold found that the significance remained in studies with LMR threshold<3.5 (HR: 2.09; 95% CI: 1.13, 3.87; P = 0.02) but disappeared in studies with LMR threshold ≥ 3.5 (HR: 1.39; 95% CI: 0.93, 2.07; P = 0.11) (**Figure 5**). Besides, subgroup analysis based on treatment methods found that the prognostic value of LMR was significant for both patients who received PD-1/PD-L1 inhibitors (HR: 4.52; 95% CI: 1.68, 12.17; P = 0.003) and those who did not receive PD-1/PD-L1 inhibitors (HR: 1.53; 95% CI: 1.11, 2.13; P = 0.01) (**Figure 6**).

### PFS

**Figure 7:**
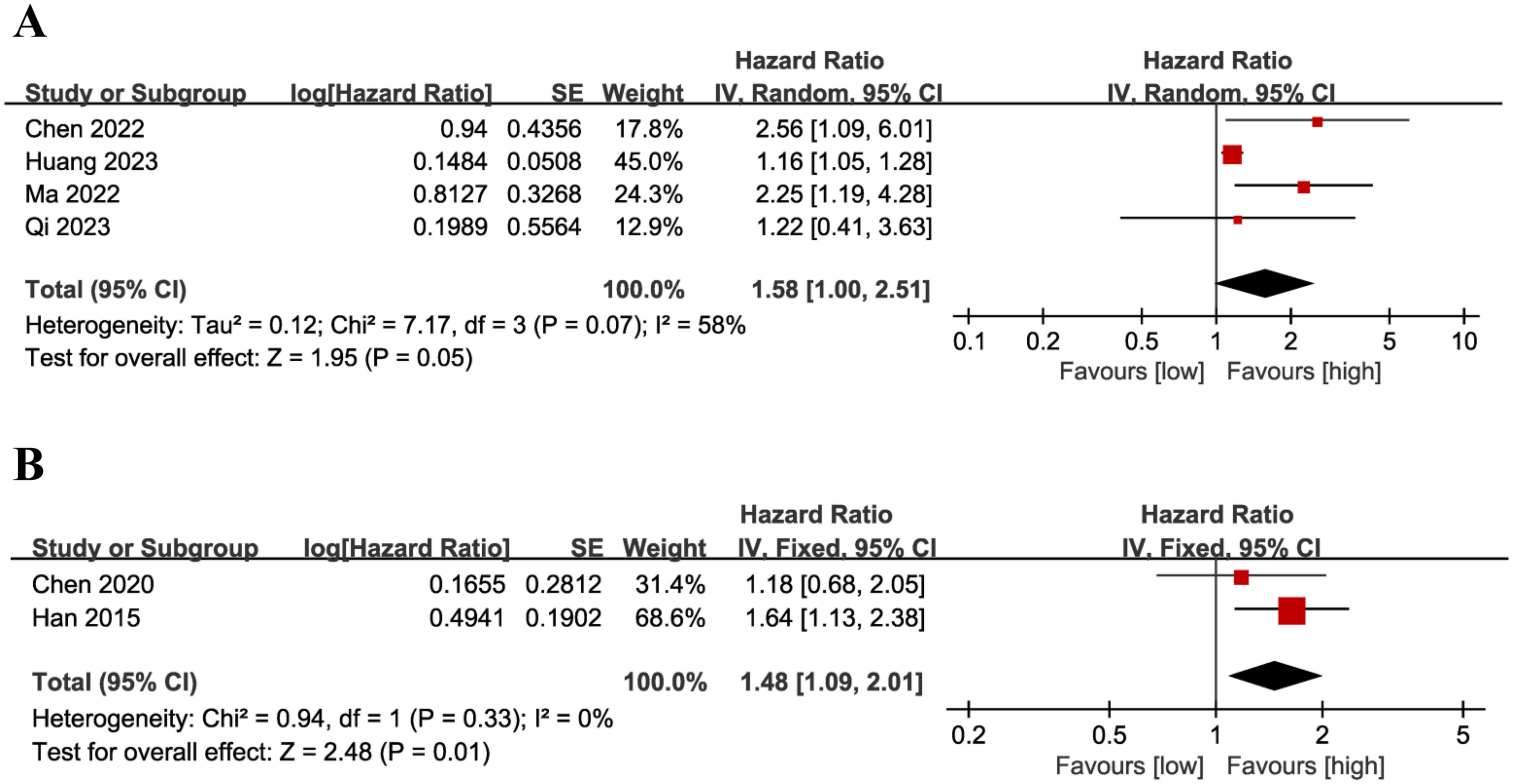
Forest plots of PFS **(A)** and DFS **(B)**.

Results of PFS were synthesized from 4 cohort studies (13–16), and meta-analysis revealed a similar PFS in the two groups (HR: 1.58; 95% CI: 1.00, 2.51; *P* = 0.05). A significant heterogeneity was observed (*I* ^2^ = 58%, *P* = 0.07) (**Figure 7A**). Results of the fixed-effects model outcome were provided in the **Supplementary File**.

### DFS

Results of DFS were synthesized from 2 cohort studies (20, 26), and meta-analysis revealed a significantly shorter DFS in the group with low LMR compared with the group with high LMR (HR: 1.48; 95% CI: 1.09, 2.01; *P* = 0.01). No significant heterogeneity was observed (*I* ^2^ = 0%, *P* = 0.33) (**Figure 7B**).

### RFS

Only one study reported the results of PFS, so we were unable to conduct a quantitative analysis. Shang et al. published a retrospective cohort study in 2020 that included 1,978 patients who underwent radical esophagectomy (19). The results suggested that RFS did not differ significantly between the low and high LMR groups (HR: 1.17; 95% CI: 0.93, 1.46; *P* = 0.18).

### Publication bias and sensitivity analysis

**Figure 8:**
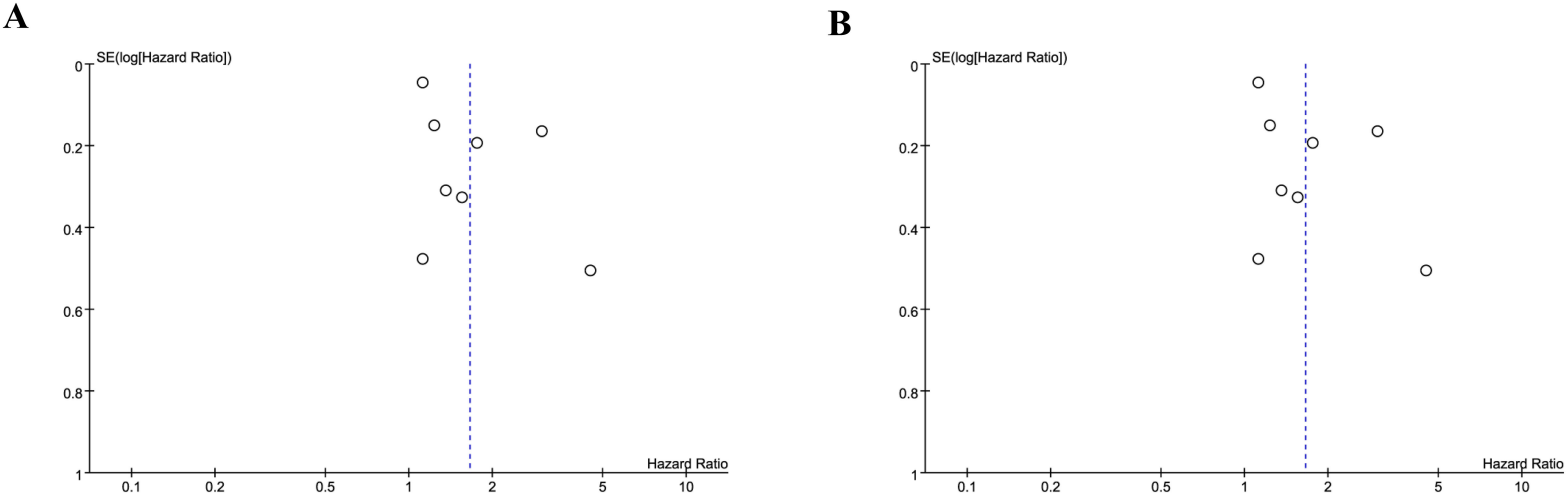
Funnel plots of OS **(A)** and PFS **(B)**.

**Figure 9:**
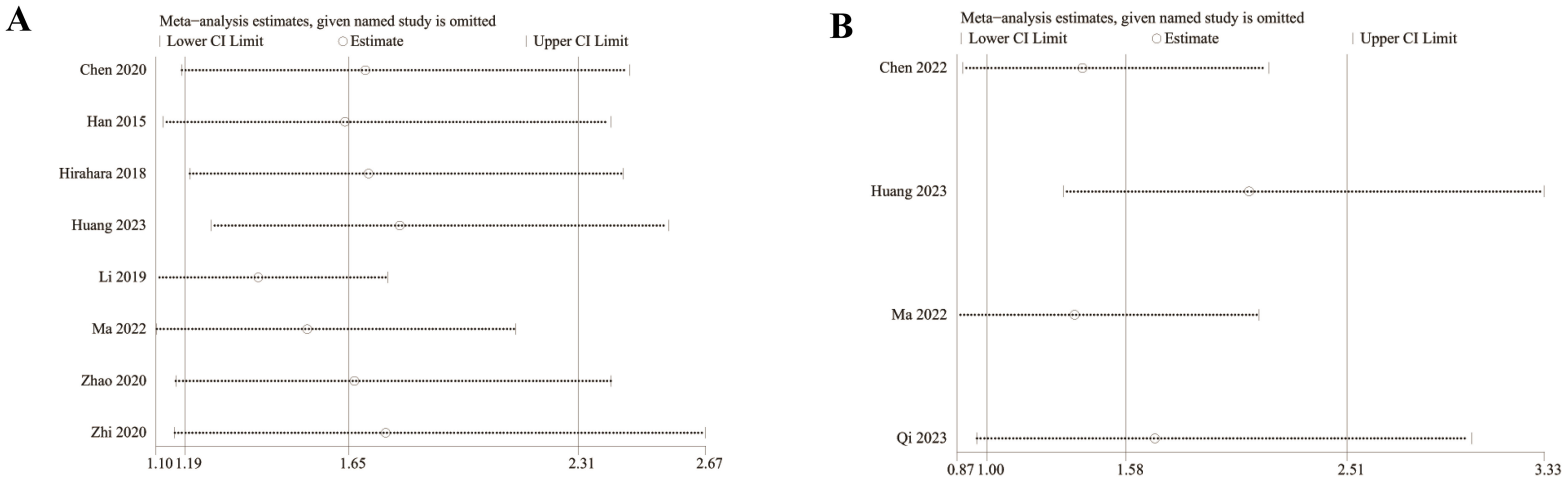
Sensitivity analysis of OS **(A)** and PFS **(B)**.

We assessed the potential publication bias through funnel plots and Egger’s regression tests for OS and PFS. No statistical (Egger’s test) or visual (funnel plots) evidence of publication bias was detected for OS (Egger’s test *P* = 0.101) (**Figure 8A**) and PFS (Egger’s test *P* = 0.188) (**Figure 8B**). In addition, we performed sensitivity analysis for the results of OS and PFS to assess the effect of each cohort study on the total HR via excluding eligible cohort studies one by one. Sensitivity analysis found that the new total HR kept stable after removing of each cohort study for OS (**Figure 9A**). However, when we excluded the data reported by Huang et al. in 2023 (14), the difference of PFS changed from non-significant to significant (**Figure 9B**).

## Discussion

The role of systemic inflammatory response in tumor is not completely clear, and systemic inflammatory response can promote or inhibit the occurrence and progression of tumor, and even affect patients’ responsiveness to systemic anti-tumor therapy (29). In addition, tumor microenvironment has been further confirmed to increase the probability of tumor metastasis, thus accelerating the progression of patients’ disease (30). The human blood system contains a variety of inflammatory response cells. According to relevant studies (26, 28, 31, 32), neutrophils, lymphocytes, monocytes and platelets in the blood are effective prognostic factors for some patients with malignant tumors. Based on the effect of these inflammatory response cells on the tumor, systemic inflammatory markers can be used to evaluate the efficacy of tumor patients. But its role in esophageal cancer remains controversial. Further systematic study of these prognostic factors through meta-analysis is conducive to evaluating the prognosis of patients.

In this meta-analysis, we evaluated the prognostic value of LMR in patients with esophageal cancer. Our results revealed a significantly shorter OS, DFS in the group with low LMR compared with the group with high LMR, suggesting that LMR has a certain predictive value for the prognosis of patients with esophageal cancer, and it should be paid attention to in the clinical treatment of esophageal cancer. In addition, subgroup analysis found that the predictive value of LMR for OS remained significant in resectable and unresectable esophageal cancers, and in studies with follow-up ≥24 months and < 24 months. However, subgroup analysis based on LMR threshold found that the significance remained in studies with LMR threshold<3.5 but disappeared in studies with LMR threshold ≥ 3.5, suggesting that it is more appropriate to limit the threshold to less than 3.5 when developing risk prediction models for esophageal cancer based on LMR.

Our findings support most of the previously published research. Studies have shown that LMR can predict the prognosis of non-elderly patients undergoing esophageal cancer surgery (32). This study analyzed the LMR, NLR and PLR of 147 patients undergoing esophageal cancer surgery, and divided the patients into elderly patients and non-elderly patients. Among the non-elderly patients, univariate analysis showed that TNM stage, tumor size, low LMR and high PLR were associated with poor prognosis. In older patients, TNM stage was the only risk factor for poor prognosis, and LMR was associated with cancer-specific survival (CSS) after resection of esophageal cancer. Low LMR, in particular, is a significant and independent predictor of poor survival in non-elderly patients. For patients with esophageal cancer, LMR can serve as a new predictor of postoperative cancer-specific survival and OS, and may help identify patients with poor prognosis even after radical resection of esophageal cancer (32). Han et al. (26) investigated the NLR, PLR and LMR of 218 patients with esophageal cancer, and found that only preoperative LMR was a prognostic factor for PFS in patients with esophageal cancer through multi-factor analysis. In addition, LMR and NLR can also be used to predict OS in patients with middle and advanced esophageal cancer after concurrent chemoradiotherapy (28), and low LMR is associated with poor prognosis in patients with esophageal cancer (31).

At present, the exact mechanism of the prognostic value of LMR in patients with esophageal cancer is not clear, and may be related to the following aspects. Firstly, as an important part of host immunity, lymphocytes play an important role in anti-tumor immune response by inducing cytotoxic cell death, inhibiting tumor cell proliferation and migration (33). Previous studies have shown that tumor-infiltrating lymphocytes are associated with good prognosis in patients with various cancers. The infiltration of CD4+ and CD8+T cells is the basis of anti-tumor immune response and induces tumor cell apoptosis through interaction (34). However, the systemic inflammatory response of tumor cells can cause immunosuppression and evade the host immune surveillance at the same time. Low lymphocyte counts are found in many human tumor tissues, and it is often associated with poorer clinical outcomes, possibly due to the fact that low lymphocyte counts may lead to an inadequate immune response (35). On the other hand, monocytes are also involved in the occurrence of tumors. More and more evidence show that tumor-related macrophages derived from monocytes exist in tumor tissues in large numbers, and macrophages promote tumor angiogenesis and anti-immune response by releasing TNF-α, vascular endothelial growth factor and epidermal growth factor (35), ultimately leading to tumor progression. Zhu et al. (36) analyzed the ratio of CD4 T cells, CD68 macrophages, CD8 T cells/CD68 macrophages and CD45RO T cells/CD68 macrophages and the prognosis of esophageal cancer patients, and the results of multivariate analysis showed that esophageal cancer patients with low CD45RO/CD68 ratio had poor DFS and OS. The CD4/CD68 and CD8/CD68 ratios were not associated with the prognosis of esophageal cancer.

However, we must acknowledge several limitations of this meta-analysis. First, due to the natural history of clinical research, only observational studies, including cohort studies and case-control studies, were included in this meta-analysis. It is well known that potential confounders and risk of bias are the greatest drawbacks of observational studies. These limitations will hopefully be addressed in future well-designed, large-sample prospective cohort studies. Secondly, all of the literature included in this meta-analysis comes from Asia, and there is a lack of data from Europe, America, and Africa. Therefore, it is unclear whether the findings of this study can be generalized to other regions. In addition, due to insufficient original data, we were unable to extract the survival information of individual patients for data merging, but could only directly extract the HR and 95% CI of the survival variables from the original studies, which may have a certain degree of bias risk. Finally, there is significant heterogeneity in some outcomes in this study. However, we aimed to explore possible causes of heterogeneity through sensitivity analysis and subgroup analysis. However, we cannot conduct sensitivity analysis and subgroup analysis for DFS and PFS due to too small sample size, which is also one of the limitations of this meta-analysis. Despite the above limitations of this article, this study is the latest and largest to report the value of LMR in predicting prognosis of patients with esophageal cancer. The findings of this study support the importance of paying attention to the level changes of LMR in the clinical treatment of esophageal cancer patients, and building a more valuable esophageal cancer prediction model based on inflammatory indicators including LMR to improve the prognosis and quality of life of esophageal cancer patients.

## Conclusion

In summary, low LMR is associated with poor prognosis in patients with esophageal cancer. Due to the simple availability and low cost of routine blood tests in clinical practice, LMR can be widely used to assess prognosis and construct risk prediction models for patients with esophageal cancer. Considering the limitations of retrospective studies, population selection bias, and significant heterogeneity, more large-scale, multicenter, prospective clinical studies are needed to further validate the relationship between LMR and prognosis of esophageal cancer.

## Data Availability

The original contributions presented in the study are included in the article/**Supplementary Material**. Further inquiries can be directed to the corresponding author.
