# Supplementary figures and images for: Prognostic value of lymphocyte to monocyte ratio in patients with esophageal cancer: a systematic review and meta-analysis
Source: Front Oncol. 2024 Nov 26;14:1401076. doi: 10.3389/fonc.2024.1401076 (PMC11628381; doi:10.3389/fonc.2024.1401076)

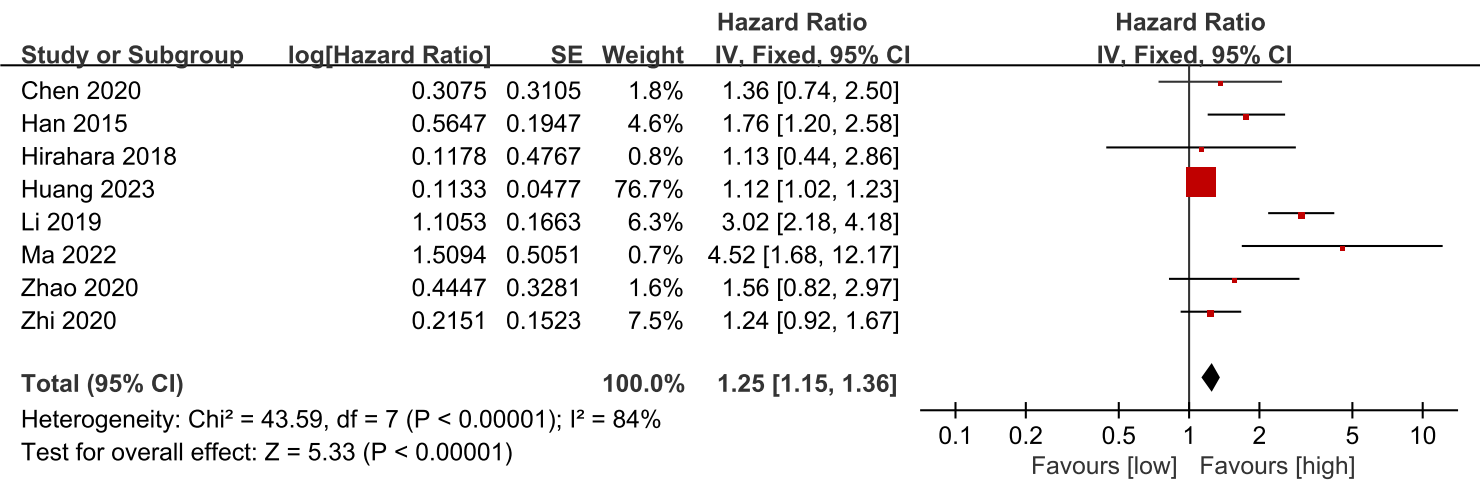

Supplement: Supplementary file 1 [file DataSheet1.zip › Forest plots of OS (fixed-effect).pdf]

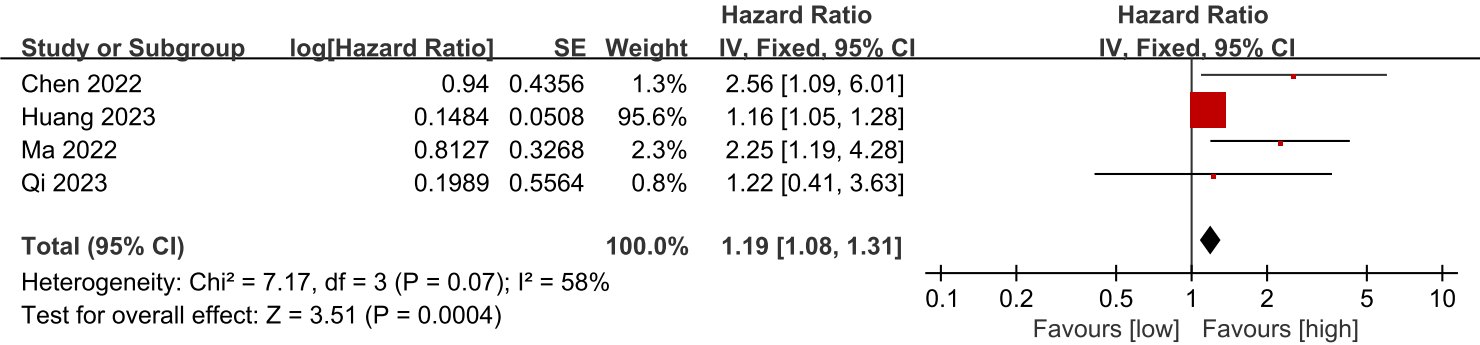

Supplement: Supplementary file 1 [file DataSheet1.zip › Forest plots of PFS (fixed-effect).pdf]
